# The Supply Chain Workforce: The Foundation of Health Supply Chains

**DOI:** 10.9745/GHSP-D-24-00444

**Published:** 2025-05-09

**Authors:** Dominique Zwinkels, Lloyd Matowe, Domina Asingizwe, Andrew N. Brown, Jonathan Moody

**Affiliations:** a People that Deliver/UNICEF, Copenhagen, Denmark.; b Eden University School of Pharmacy, Lusaka, Zambia.; c University of Rwanda, Kigali, Rwanda.; d Management Sciences for Health, Canberra, Australia.; e People that Deliver, Copenhagen, Denmark.

## Abstract

The articles in this supplement delve into the most pertinent topics in human resources for supply chain management (SCM), offering case studies and insights that exemplify best practices to strengthen the capacity of the health SCM workforce, support well-functioning health systems and increase access to health commodities.

## HUMAN RESOURCES FOR SUPPLY CHAIN MANAGEMENT: ESSENTIAL TO A WELL-PERFORMING HEALTH SYSTEM

The global health community has agreed to accelerate efforts to achieve universal health coverage (UHC) by 2030, as nearly a third of the world’s population does not have access to quality health services, nor access to safe, effective, and affordable essential medicines and vaccines (Sustainable Development Goal 3.8).^1^ UHC, though, requires an efficient and well-performing health system that provides the entire population with access to quality services, health workers, medicines, and technologies. For a country to achieve UHC, well-trained, motivated, and supported health workers are needed.^2^

Health care supply chain (SC) functions in low- and middle-income countries (LMICs), including the forecasting, procurement, storage, and distribution of health products, are provided by a variety of workers, including pharmacists, doctors, nurses, technicians, logisticians, and warehouse and transport personnel, who are often unqualified and unskilled for the purpose. These crucial functions, which are required for the delivery of health products, technologies, and associated services, are sometimes overlooked and not given the necessary attention by national governments. In many LMICs, health SCM is not a recognized profession.

In LMICs, there is a shortage of appropriately qualified SC professionals. Recent figures show that the global health workforce shortage is declining—from 18 million in 2013 to 15 million in 2020 and projected to be 10 million by 2030; however, progress is slower in some regions, including Africa and the Eastern Mediterranean.^3^ The World Health Organization (WHO) supports strengthening the capacities of SCs and foresees that roles related to procurement and supply chain management (SCM) will require increased numbers of workers and the creation of new roles in this sector.^4^ Strengthening workforce capacities is one of the primary perceived needs indicated by WHO’s second round of the national pulse survey of 135 countries. ^5^ Health outcomes in LMICs will only continue to improve if the capacity and skills of the health SC workforce are developed, enabling the access, availability, and appropriate use of quality health commodities.^6^

SC success is highly dependent on human talent; companies that invest more in the development of their SC employees achieve greater SC outcomes and organizational performance.^7^ If SCM were transformed into a recognized profession of the highest integrity and if SC professionals in LMICs were better supported to develop and apply their technical and managerial competencies, we would see improvements in SC performance and health commodities would be more readily available at service delivery points.

## OVERVIEW OF THE SUPPLEMENT

The articles in this supplement delve into the most pertinent topics in human resources (HR) for SCM, offering examples of best practices, interventions, and tools that can increase the impact of interventions and suggesting how to attract, build, and retain the skilled and motivated workforce needed to manage and transform health SCs.

Zwinkels et al.^8^ explain the reasons for People that Deliver (PtD)’s founding and make the point that the coalition plays an irreplaceable role in convening a wide array of stakeholders in the sector, advocating continued investments in HR for SCM and creating tools and resources that help countries develop and sustain their health SC workforce.

Meier et al.^9^ describe how Rwanda used the theory of change (TOC) to assess the HR in its health SC and develop the capacity of its SC workforce. Steele et al.^10^ discuss the application of the TOC for building HR for SCM in multiple countries and identify the elements necessary for successful implementation of the TOC.

Brown et al.^11^ present PtD’s SCM professionalization framework as a solution to help countries professionalize the health SC workforce. By clearly defining the pathways for developing competencies across different professional designations, this 4-part framework offers a blueprint for aligning education, training, and workforce development efforts to effectively meet health SCM needs.

Bobo et al.^12^ present the STEP 2.0 program as a potential solution to the persistent leadership problems in many countries. STEP 2.0 is at the forefront of a concerted effort to draw attention to the required leadership and management competencies for health SCM and is pivotal to professionalizing the SC workforce.

Truog et al.^13^ present original research on the barriers women face in entering the health SC profession and make recommendations to improve gender equity in the SC workforce.

Duwiejua et al.^14^ identify HR for SCM initiatives that have the potential to be scaled up. The featured innovative and targeted interventions have demonstrated their ability to enhance HR capacity and transform SC performance, indicating that real benefits can be achieved by using “promising practices.”

Msimuko et al.^15^ highlight the importance of creating opportunities for young people in the SC industry and suggest how to better attract and retain them in the profession.

We believe the articles in this supplement convey the importance of developing and professionalizing the workforce required for health SCs and provide new insights to help further develop its capacity.

## RECOMMENDATIONS TO ENSURE SUPPLY CHAIN MANAGEMENT PROFESSIONALIZATION

As interest in HR for SCM continues to grow, there has been a proliferation of organizations and technical partners involved in building the health SCM workforce and raising awareness of the need for increased investments. Enhanced coordination and collaboration among all stakeholders will be necessary to effectively align donor and government investments. Additionally, ensuring country-level commitment to HR for SCM remains a challenge in the context of competing health priorities in a restricted fiscal space.

We should aim to develop SCM systems and workforces that are agile, robust, and designed to evolve. This will be essential as health-seeking behaviors change over time, owing to the impact of external factors, including migration, demographic transitions, and technology. We make the following suggestions on how to attract, build, and retain a skilled and motivated workforce needed to manage and transform health SCs.

### Invest in Workforce Development

SCM professionalization and effective leadership should be 2 priority areas for countries, while increased investments in staffing, skills, motivation, and working conditions will be required to ensure the availability and use of medicines. This is essential to achieving UHC. Findings from PtD’s business case for investment in HR for health SCM suggest that investments in HR for SCM offer value for money.^16^ These investments will be required of donor organizations, private-sector companies, and countries themselves as they transition away from donor support and focus on strengthening their health systems.

### All Stakeholders—Including Governments, Educational Institutions, and the Private Sector—Must Collaborate

In the coming years, the role of governments, academia, and professional associations will be as important as ever, especially as donor funding decreases and SCM professionalization is embedded within national systems and organizations. Stakeholders must work together to ensure that investments and activities—in particular, private-sector investments in pre-service educational programs—are aligned with country priorities to stimulate further investment in the SCM field.

### Develop Policies That Encourage Women and Youth to Join and Remain in the Profession

Retention and performance remain issues of concern, with high levels of turnover and the loss of public-sector staff to the private sector compounding staffing shortages in rural areas.^17^ Encouraging underrepresented groups into the profession, such as youth and women, and supporting them in all areas of the workplace can help to address this shortage.

### Develop Capacity in Data Science, Analytics, Outsourcing, Contracting, and Monitoring

There is a need to develop capacity in data science (including artificial intelligence), analytics, outsourcing, contracting, and monitoring of SC performance across the public and private sectors. The digital transformation in SCM will accelerate innovation and transform the SC into a core strength for organizations.

## Notes

**Peer Reviewed**
